# Awareness and knowledge about HPV and primary HPV screening among women in Great Britain: An online population-based survey

**DOI:** 10.1177/09691413231205965

**Published:** 2023-10-24

**Authors:** Jo Waller, Frances Waite, Laura Marlow

**Affiliations:** Cancer Prevention Group, School of Cancer & Pharmaceutical Sciences, 4616 King's College London, London, UK

**Keywords:** Knowledge, understanding, HPV primary screening, cervical screening, screening intervals

## Abstract

**Objectives:**

Human papillomavirus (HPV) primary testing for cervical screening is being implemented around the world. We explored HPV awareness, and knowledge about primary screening in Great Britain (England, Scotland and Wales), where it has been in place for several years, ahead of extended screening intervals being implemented in England.

**Setting/Methods:**

Women aged 18–70 (*n* = 1995) were recruited by YouGov from their online panel in August 2022. The weighted sample (*n* = 1930) was population-representative by age, region, education and social grade. We measured HPV awareness, knowledge (excluding those unaware of HPV) using eight true/false items, and understanding of the role of HPV testing in cervical screening.

**Results:**

Overall, 77.6% (1499/1930) of women were aware of HPV. When asked to identify the statement describing how cervical screening works, only 12.2% (236/1930) correctly selected the statement reflecting HPV primary screening (13.5% (194/1436) in screening-eligible women). Excluding those unaware of HPV, most participants had heard about the virus in the context of cervical screening (981/1596; 61.5%) or HPV vaccination (1079/1596; 67.6%). Mean knowledge score was 3.7 out of 8 (SD = 2.2) in this group. Most knew that an HPV-positive result does not mean a woman will definitely develop cervical cancer (1091/1499; 72.8%) but far fewer were aware of the long timeline for HPV to develop into cervical cancer (280/1499; 18.7%).

**Conclusions:**

Only three-quarters of women in Britain are aware of HPV, and knowledge of primary screening is very low, even among screening-age women. This points to continued need for awareness-raising campaigns to ensure informed choice about screening and mitigate public concern when screening intervals are extended.

## Introduction

Cervical screening in England, Scotland and Wales now uses human papillomavirus (HPV) testing as the primary screening test (henceforth referred to as ‘HPV primary screening’). This means screening samples are first tested for the presence of high-risk HPV and only if HPV is found is the sample checked for cellular abnormalities using cytology.^1^ HPV primary screening was fully rolled-out in England in 2019 (following a pilot in several areas from 2013), Scotland in 2020 and Wales in 2018.^2–4^ This new screening algorithm paves the way for extending cervical screening intervals as HPV testing has a better negative predictive value than cytology-based screening.^5^ When such an interval change (from 3 to 5 years among women aged 25–49 years) was announced in Wales in January 2022, the public backlash suggested awareness of the new HPV-based screening was low.^6^ This is consistent with data collected using the generic Cancer Awareness Measure in 2014 and 2017 which found that only 0.1% of adults named HPV (unprompted) as a risk factor for cancer.^7^ Recent work suggests that interval extensions are more acceptable once the rationale (based on HPV primary screening) is understood.^8–10^

In addition to public messaging more broadly, HPV awareness and knowledge are important for the individuals being offered screening. In order to make an informed choice about participation, the eligible population needs to: (a) be aware of HPV; and (b) understand that everyone taking part in screening will now have an HPV test. In addition, certain aspects of HPV knowledge have been found to be important in mitigating the adverse psychological impact of receiving an HPV-positive result.^11–13^ These include the fact that HPV is common, that it does not have symptoms, that a positive HPV result does not mean someone will definitely develop cancer, and that the timeline from HPV infection to cancer development is long. Previous research has found that awareness and knowledge of HPV are still relatively low in the general population and recent qualitative studies suggest women receiving HPV-positive screening results have a number of concerns and unanswered questions.^13,14^ This uncertainty is thought to contribute to observed short-term anxiety among HPV-positive women.^15^

This study aimed to answer the following questions:

1. What proportion of women in Great Britain are able to name HPV as a risk factor for cervical cancer?
2. What proportion of women are aware of HPV (have heard of it, when prompted) and in what context?
3. Are women able to recognise a description of HPV primary screening as being the way that screening is currently carried out in Britain?
4. What are the levels of knowledge about key aspects of HPV among those who are aware of it?
5. Are there demographic differences in HPV awareness or in HPV knowledge among those who have heard of it?

## Methods

### Design

A cross-sectional, population-based online survey was carried out by YouGov on behalf of the research team, using YouGov's opt-in online panel. Questions developed for the study were added to YouGov's daily Great Britain omnibus survey between 11 and 15 August 2022. Participants are recruited onto YouGov's online research panel via the YouGov website and through adverts on social media channels. Participants receive points for taking part in surveys; once a certain threshold is reached, points can be exchanged for shopping vouchers. There are over 2.7 million people on the YouGov's UK panel. Stratified random sampling is used to select 2000 adults, who are representative of the British population with respect to age, gender, region and social grade, for each daily omnibus survey.

### Participants

Women aged 18–70 years were eligible to take part. This age range was selected to include adults who would soon be, or had until recently been, eligible for cervical screening. We aimed to recruit a sample of approximately 1800 by running the questions in two surveys. This number was selected primarily based on funding considerations, but we estimated that if 55% of respondents were aware of the link between HPV and cervical cancer, based on a previous study,^16^ a sample size of 1800 would give us 95% confidence intervals of +/-2.3% around this estimate.

### Measures

The full survey is available on Open Science Framework (OSF) along with an outline protocol for the study (https://osf.io/58hdf/). We assessed awareness of HPV using unprompted and prompted questions, the context within which people had heard about HPV, understanding about HPV primary screening methods and knowledge of different aspects around HPV.

Unprompted awareness of HPV as a risk factor for cervical cancer was assessed using an open question taken from the Cervical Cancer Awareness Measure:^17^ ‘What things do you think affect a woman's chance of developing cervical cancer?’ Open responses were typed in by participants and coded by FW (see Supplemental materials for description of coding). Participants who mentioned the terms *HPV* or *human papillomavirus* in their response (including *HPV vaccination*) were coded ‘HPV mentioned’, all other participants were coded ‘HPV not mentioned’.

Prompted awareness of HPV was assessed with the question: ‘Have you ever heard of human papillomavirus, or HPV?’ (Yes, I have; No, I have not; Don’t know/Can’t recall).^16^ Those who responded Yes or Don’t know were also asked to indicate which of six options represented the context in which they had heard of HPV: Cervical Screening; HPV vaccination; Cancer in general; Sexual health; Other; Don’t know/Can’t recall (multiple options could be selected).

We developed an item designed to assess whether participants could correctly identify how screening samples are tested (i.e. in line with HPV primary screening). Participants were asked ‘As far as you know, which ONE of these statements best describes how cervical screening works?’ with the response options: All samples are checked for cell changes and sometimes they are also tested for HPV; All samples are tested for HPV and sometimes they are also checked for cell changes (considered correct); All samples are tested for HPV and checked for cell changes; Don’t know. The order of response options was randomised to avoid ordering effects. Responses were dichotomised into correct vs. incorrect/don’t know.

Participants who had heard of HPV (or didn’t know if they had) were also presented with eight true/false items (with a ‘don’t know’ option). These items were taken from previous studies and were designed to assess aspects of HPV knowledge that have been shown to be important in understanding psychological responses to an HPV-positive result as well as acceptability of longer screening intervals;^9,18,19^ see Table 1 for full wording. The order of items was randomised. An overall knowledge score was calculated by summing the eight items (one point for each correct response; possible range 0–8).

**Table 1.** Descriptives of HPV awareness and knowledge questions (weighted, unadjusted).

	*n*	% (95% CI)
**Causes of cervical cancer, unprompted (*n* = 1930)**		
HPV mentioned	263	13.6 (12.2–15.2)
HPV not mentioned	1667	86.4 (84.8–87.8)
**Heard of HPV (*n* = 1930)**		
Yes, I have	1499	77.6 (75.7–79.4)
No, I have not	334	17.3 (15.7–19.1)
Unsure	97	5.0 (4.2–6.1)
**HPV primary screening (*n* = 1930)**		
Correct understanding	236	12.2 (10.8–13.7)
Incorrect understanding	1113	57.7 (55.5–59.8)
Not sure	581	30.1 (28.1–32.2)
**Context heard of HPV^a^ (*n* = 1499)**		
HPV vaccination	1065	71.1 (68.7–73.3)
Cervical screening	966	64.4 (62.0–66.8)
Sexual health	703	46.9 (44.4–49.4)
Cancer in general	391	26.1 (24.0–28.3)
Other	43	2.9 (2.1–3.9)
Not sure	88	5.9 (4.8–7.1)
**Knowledge statements^b^ (*n* = 1499)**		
If a woman tests positive for HPV, she will definitely get cervical cancer (F)	1091	72.8 (70.5–75.0)
HPV always has visible signs or symptoms (F)	1046	69.8 (67.5–72.1)
HPV is very rare (F)	939	62.7 (60.2–65.1)
HPV can be passed on by genital skin-to-skin contact (T)	724	48.3 (45.8–50.8)
An HPV test can tell you how long you have had an HPV infection (F)	581	38.7 (36.3–41.2)
If an HPV test shows that a woman does not have HPV, her risk for of cervical cancer is low (T)	494	32.9 (30.6–35.3)
Most sexually active people will get HPV at some point in their lives (T)	342	22.8 (20.8–25.0)
An HPV infection can develop into cervical cancer very quickly (F)	280	18.7 (16.8–20.7)

^a^ Multiple options could be selected.

^b^ Proportion answering statements correctly. T = True; F = False.

Demographic characteristics included age, occupational social grade (using Office for National Statistics 2011^20^ and the National Readership Survey 2016 classification),^21^ ethnic group, work status, marital status, region of residence, presence of children in the household and parent/guardian status. These were assessed with YouGov's standard questions. The National Readership Survey Social Grade Classification includes five categories based on occupation of the chief income earner in the household as follows: AB = Higher and intermediate managerial, administrative, professional occupations; C1 = Supervisory, clerical and junior managerial, administrative, professional occupations; C2 = Skilled manual occupations; DE = Semi-skilled and unskilled manual occupations, Unemployed and lowest grade occupations. We also assessed self-reported cervical screening status and HPV vaccine status. Screening-eligible women (aged 25–64) were classified as ‘up-to-date’, ‘overdue’ or ‘never screened’. All women aged 25–64 years reporting screening within the last 3 years were coded as up-to-date. Women screened within the last 3–5 years were considered up-to-date if they lived in Wales or Scotland or if they lived in England and were aged 50–64, in line with national screening recommendations.

### Analyses

YouGov provided a quality-checked dataset including sample weights, allowing us to correct for sampling bias in relation to age, region, education and social grade. All reported analyses were carried out by the King's College London research team using the SPSS v27 Complex Samples function, allowing us to incorporate sampling weights. The weighted sample was representative of adult women in Britain with respect to age, region, education and social grade. Descriptives for each of the outcome variables are presented using proportions and means, with 95% confidence intervals. Some descriptives have been reported for two sub-groups: ‘Screening-eligible participants’ (defined as those aged 25–64 years) and ‘HPV vaccine cohort’ (defined as those under 30 years who would have been offered HPV vaccination in the school-based programme). Unadjusted logistic regression analyses were used to explore predictors of: (a) awareness of HPV as a risk factor for cervical cancer; (b) having previously heard of HPV; and (c) being aware of HPV primary screening. Unadjusted general linear models were used to examine predictors of higher HPV knowledge score. Adjusted models are included as Supplementary Tables S1 and S2. Due to the large number of analyses, we used *p* < .01 as a conservative estimate of statistical significance.

## Results

### Sample characteristics

Overall, 1995 women completed the survey (weighted *n* = 1930). Weighted and unweighted characteristics of the sample are shown in Table 2. Mean age of the sample was 45 years (SD: 15 years), 60.5% were working (full- or part-time) and 64.6% were from a White ethnic background. In the HPV vaccine cohort, 67.3% reported having received HPV vaccination and 68.2% of the screening-eligible cohort were considered up-to-date with cervical screening based on self-report.

**Table 2.** Sample characteristics.

	Weighted (*n* = 1930)	Unweighted (*n* = 1995)
**Age in years, mean (SD)**	45.02 (15.37)	45.39 (15.35)
**Age group, *n* (%)**		
18–24	230 (11.9)	220 (11.0)
25–34	351 (18.2)	366 (18.3)
35–44	381 (19.7)	395 (19.8)
45–54	318 (16.5)	327 (16.4)
55–64	386 (20.0)	396 (19.9)
65–70	265 (13.7)	291 (14.6)
**Occupational social grade, *n* (%)**		
AB (high)	494 (25.6)	538 (27.0)
C1	598 (31.0)	630 (31.6)
C2	415 (21.5)	375 (18.8)
DE (low)	423 (21.9)	452 (22.7)
**Ethnic background, *n* (%)**		
Any White background	1247 (64.6)	1297 (65.0)
Mixed ethnic background	25 (1.3)	26 (1.3)
Any Asian background	48 (2.5)	47 (2.4)
Any Black background	18 (0.9)	17 (0.9)
Other	4 (0.2)	4 (0.2)
Prefer not to say/chose not to answer	588 (30.4)	604 (30.3)
**Work status, *n* (%)**		
Working full time	758 (39.3)	787 (39.4)
Working part time	413 (21.4)	420 (21.1)
Full-time student	109 (5.6)	106 (5.3)
Retired	313 (16.2)	337 (16.9)
Unemployed	94 (4.9)	93 (4.7)
Not working or other	244 (12.6)	252 (12.6)
**Marital status, *n* (%)**		
Married/civil partnership/living as married	1077 (55.8)	1120 (56.1)
Separated/divorced/widowed	237 (12.3)	250 (12.5)
Never married	613 (31.8)	621 (31.1)
Chose not to answer	4 (0.2)	4 (0.2)
**Region/nation, *n* (%)**		
England	1659 (85.9)	1711 (85.8)
Wales	93 (4.8)	96 (4.8)
Scotland	179 (9.2)	188 (9.4)
**Children in household, *n* (%)**		
No	1370 (71.0)	1423 (71.3)
Yes	560 (29.0)	572 (28.7)
**Screening status – eligible only^a^, *n* (%)**		
Up to date	979 (68.2)	1012 (68.2)
Overdue	268 (18.7)	277 (18.7)
Never screened	88 (6.1)	92 (6.2)
Don’t know/can’t recall	50 (3.5)	51 (3.4)
Prefer not to say/chose not to answer	50 (3.5)	52 (3.5)
**HPV vaccine status – HPV vaccine cohort only^b^, *n* (%)**		
Had the vaccine	288 (67.3)	290 (68.1)
Not had the vaccine	76 (17.7)	76 (17.8)
Don’t know/can’t recall	45 (10.6)	42 (9.9)
Prefer not to say/chose not to answer	19 (4.4)	18 (4.3)

*Notes:* The survey was carried out online, 11–15th August 2022. Weighted analyses are representative of all GB female adults (aged 18+).

^a^ Screening eligible includes those aged 25–64 (weighted = 1436; unweighted *n* = 1484).

^b^ HPV vaccine cohort: those aged 30 or below (weighted = 427; unweighted *n* = 426).

### HPV awareness

In response to the unprompted question assessing awareness of cervical cancer risk factors, 13.6% of participants mentioned HPV (including if it was mentioned in relation to the HPV vaccine). When asked explicitly about whether they had heard of HPV, 77.6% said yes, 17.3% said no and 5.0% were unsure (see Table 1).

Among participants who had heard of HPV (*n* = 1499), 71.1% had heard about HPV in the context of HPV vaccination (84.3% among the HPV vaccine cohort) and 64.4% had heard about HPV in the context of cervical screening (68.8% among the screening-eligible cohort). Across the total sample, only 12.2% of participants selected the statement that most closely represented how screening samples are tested, that is in line with HPV primary screening (13.5% in the screening-eligible cohort). Excluding those who had not heard of HPV made little difference to this (13.5% overall and 14.8% in the screening-eligible cohort).

Table 3 shows unadjusted logistic regression analyses for the three awareness outcomes. Age was significantly associated with unprompted knowledge of HPV as a risk factor for cervical cancer (*p* < .001), having heard of HPV before (*p* < .001), and understanding of HPV primary screening (*p* < .001). The youngest women (18–24 years) and the older women (50–64 and 65–70 years) were less likely to mention HPV as a cervical cancer risk factor, less likely to have heard of HPV before, and less likely to select the statement that most closely represents HPV primary screening, compared to women aged 25–49 years. Occupational social grade was associated with mentioning HPV as a cervical cancer risk factor (*p* < .001) and having heard of HPV before (*p* < .001), with a gradient from lowest awareness in the lowest social grade groups to highest awareness in the highest social grade groups. Not surprisingly in the HPV vaccine cohort those who had been vaccinated had higher previous awareness of HPV (86.1% vs. 59.5%, *p* < .001) and were more likely to mention HPV as a risk factor for cervical cancer without prompting (17.7% vs. 9.9%, though this difference was not significant *p* = .054). These patterns were very similar in adjusted analyses (presented in Supplementary Table S1).

**Table 3.** Predictors of HPV awareness and knowledge of HPV primary screening (weighted, unadjusted).

	HPV risk factor for cervical cancer^a^		Heard of HPV before^b^		Understanding of HPV primary screening^c^	
	% mentioned	OR (95% CI)	% yes	OR (95% CI)	% correct	OR (95% CI)
**Age**						
18–24	11.7	0.63 (0.40–0.99)	70.4	0.48 (0.34–0.68)	9.6	0.56 (0.35–0.91)
25–49	17.1	Ref	83.2	Ref	16.0	Ref
50–64	11.0	0.60 (0.44–0.82)	76.9	0.67 (0.51–0.87)	9.3	0.53 (0.38–0.75)
65–70	8.7	0.45 (0.29–0.71)	66.3	0.40 (0.29–0.53)	7.5	0.43 (0.27–0.69)
**Occupational social grade**						
AB (high)	19.3	Ref	83.8	Ref	12.6	Ref
C1	15.1	0.74 (0.54–1.00)	78.8	0.71 (0.53–0.96)	12.7	1.02 (0.72–1.44)
C2	9.9	0.45 (0.30–0.68)	76.1	0.61 (0.44–0.85)	13.3	1.07 (0.72–1.58)
DE (low)	8.7	0.40 (0.27–0.60)	70.2	0.45 (0.33–0.62)	10.2	0.80 (0.54–1.19)
**Ethnic group**						
Any white background	14.6	Ref	78.7	Ref	11.1	Ref
All other ethnicities	8.4	0.57 (0.27–1.19)	70.6	0.64 (0.40–1.02)	11.5	1.05 (0.54–2.02)
Prefer not to say/chose not to answer	12.4	0.83 (0.63–1.11)	76.7	0.89 (0.70–1.12)	14.6	1.37 (1.03–1.83)
**Region**						
England	13.6	Ref	77.3	Ref	29.4	Ref
Scotland	12.4	0.90 (0.57–1.41)	79.8	1.15 (0.79–1.68)	33.5	1.08 (0.68–1.70)
Wales	17.2	1.37 (0.79–2.36)	78.7	1.11 (0.67–1.85)	37.6	1.17 (0.64–2.14)
**Marital status**						
Married/civil partnership/living as married	13.8	Ref	79.7	Ref	13.3	Ref
Separated/divorced/widowed	13.1	0.93 (0.62–1.40)	73.3	0.69 (0.50–0.95)	8.5	0.62 (0.39–0.99)
Single, never married	13.5	0.98 (0.73–1.30)	75.9	0.80 (0.63–1.02)	11.3	0.83 (0.62–1.13)
**Cervical screening status (eligible only)**						
Up to date	16.7	Ref	83.9	Ref	15.2	Ref
Overdue	11.9	0.68 (0.46–1.01)	78.0	0.68 (0.49–0.95)	9.7	0.59 (0.38–0.91)
Never attended	11.4	0.64 (0.33–1.23)	80.9	0.86 (0.49–1.52)	9.1	0.58 (0.28–1.18)
**HPV vaccination status (HPV vaccine cohort only)**						
Not vaccinated/unsure	9.9	0.51 (0.26–1.01)	59.5	0.24 (0.14–0.40)	17.4	1.54 (0.85–2.79)
Vaccinated	17.7	Ref	86.1	Ref	12.2	Ref

CI: confidence interval.

^a^ Reference category didn’t mention HPV as a risk factor.

^b^ Reference category haven’t heard/unsure.

^c^ Reference category incorrect/don’t know.

### Knowledge of different aspects of HPV

Knowledge of specific aspects of HPV was variable (see Table 1). Most participants who were aware of HPV knew that an HPV-positive result does not mean that someone will definitely get cervical cancer (72.8%) and that HPV does not have visible signs and symptoms (69.8%), but only 22.8% knew that most sexually active people will get HPV in their lifetime and just 18.7% were aware of the long timeline from HPV infection to cervical cancer development. The mean total knowledge score was 3.7 (SD 2.2) out of a possible 8.

Table 4 shows unadjusted linear regression analyses exploring predictors of knowledge score. Knowledge scores were highest in women aged 25–49 years and there was a gradient by occupational social grade with lowest scores among women in the lowest group (DE) and highest scores in the highest group (AB). Participants from ethnic minority groups had lower knowledge scores than those from White ethnic groups. Among those who were eligible for screening, screening status was also associated with HPV knowledge score (*p* < .001) with higher knowledge scores in those who were considered up to date. These patterns were very similar in adjusted analyses (see Supplementary Table S2).

**Table 4.** Predictors of HPV knowledge score among those who had heard of HPV, *n* = 1499 (weighted, unadjusted).

	Mean (95% CI)	*p*-Value
**Age**		
18–24	3.09 (2.77–3.41)	*F*(3,1550) = 16.24, *p* < .001
25–49	4.04 (3.88–4.20)	
50–64	3.31 (3.10–3.51)	
65–70	3.44 (3.17–3.71)	
**Occupational social grade**		F(3,1550) = 3.57, *p* = .014
AB (high)	3.81 (3.62–4.01)	
C1	3.82 (3.63–4.01)	
C2	3.49 (3.23–3.76)	
DE (low)	3.41 (3.17–3.65)	
**Ethnic group**		
Any white background	3.61 (3.48–3.75)	F(2,1551) = 7.75, *p* < .001
All other ethnicities	2.90 (2.42–3.39)	
Prefer not to say/chose not to answer	3.90 (3.70–4.10)	
**Region**		F(2,1551) = 0.30, *p* = .744
England	3.69 (3.57–3.80)	
Scotland	3.57 (3.05–4.10)	
Wales	3.56 (3.22–3.90)	
**Marital status**		F(2,1548) = 0.35, *p* = .705
Married/civil partnership/living as married	3.71 (3.56–3.85)	
Separated/divorced/widowed	3.57 (3.25–3.90)	
Single, never married	3.63 (3.43–3.83)	
**Cervical screening status (eligible only^a^)**		F(2,1141) = 10.61, *p* < .001
Up to date	4.01 (3.86–4.16)	
Overdue	3.39 (3.12–3.66)	
Never attended	3.29 (2.83–3.76)	
**HPV vaccination status (HPV vaccine cohort only^b^)**		
Vaccinated	3.68 (3.40–3.95)	F(1321) = 0.21, *p* = .884
Not vaccinated/unsure	3.63 (3.13–4.14)	

CI: confidence interval.

^a^ Screening eligible includes those aged 25–64.

^b^ HPV vaccine cohort includes those aged 30 or below.

## Discussion

This study is the first to assess HPV awareness and knowledge in a broadly population-representative sample of British women since the introduction of HPV primary screening. Only about 1 in 7 women were able to spontaneously name HPV as a risk factor for cervical cancer but when prompted, almost 80% said they had heard of it. Unsurprisingly this was mainly in the cervical screening and/or HPV vaccination context. When asked about how cervical screening is done, only 12% correctly selected the statement most closely reflecting primary screening, with all samples being tested for HPV. Specific knowledge about HPV was moderate, with a mean score of 3.7 out of 8 and fewer than 75% of participants giving a correct response on any of the knowledge items. These figures only include women who had heard of HPV.

The findings provide evidence that women's awareness of HPV has increased over the last decade, as would be expected with school-based vaccination having been in place for 15 years, and HPV testing increasingly being used in screening. In a 2013 study, 62% of UK women had heard of HPV compared to 78% in the present study.^22^ However, there is less evidence of any increase in specific knowledge that might support understanding of the rationale for HPV primary screening or interpretation of an HPV-positive result. The proportions of women getting the knowledge items correct were similar to, and sometimes lower than for the same items in 2013.^19,22^ Of particular concern was the finding that just over 20% of women were aware that most sexually active people will contract HPV at some point in their lives, slightly lower than was found in 2013.^22^ Awareness of the high prevalence of the virus is likely to reduce stigma and adverse psychological outcomes^12^ in the huge numbers of women now testing positive at screening. More reassuring was the increase in the proportion of women who knew that an HPV-negative result indicates low risk of cervical cancer from 24%^19^ to 33%; though there is still a need to increase this further.

Ensuring that there is good public understanding of HPV primary screening is also important in the context of a proposed move from 3 to 5 yearly screening intervals for younger women in the English programme. If the rationale for this change is poorly communicated, there is likely to be resistance from the public and risk of a loss of confidence in the programme.^23^ Part of the rationale for extended intervals is the long time it takes for HPV to develop into cervical cancer, and this was poorly understood even by women who had heard of HPV in our study. HPV primary screening appointments could be a useful opportunity to check understanding and provide information, increasing knowledge in the run-up to the interval change in England.

HPV primary screening in now in place in England, Wales and Scotland, but the majority of women seem unaware of this change. Only 12% identified the statement that ‘All samples are checked for HPV and sometimes they are also checked for cell changes’ as most closely representing how cervical screening works, with 30% responding ‘Don’t know’ and others incorrectly believing that all samples are checked for cell changes. This low awareness may be due, in part, to some participants, not yet having received an invitation for HPV primary screening since its implementation (particularly those aged under 25 or on 5-year screening intervals). However, the proportion aware of HPV primary screening was only 16% in the 25–49 year age group, most of whom would have been invited for HPV primary screening between 2019 and our survey in 2022. Previous work has shown that women taking part in HPV testing frequently have unmet information needs^14^ and our findings suggest that women may not be making an informed choice about screening participation. Receiving an HPV-positive result can engender short-term anxiety, concerns about relationships and confusion;^15,19,24^ and this is likely to be more pronounced in women who were not expecting to have an HPV test as part of screening. Raising awareness of HPV primary screening should be an urgent priority, and health professionals delivering screening may be best placed to ensure those taking part are informed about HPV testing and to signpost them to further information.

The socio-demographic patterns in this study reflect previous surveys that highlight lower HPV awareness in younger and older women than those of middle age, and in those from lower compared with higher socio-economic groups.^25,26^ We also found that inequalities extend beyond awareness. Among participants who had heard of HPV, we found lower levels of knowledge in younger women, those from lower social grades, and those from ethnic minority backgrounds. However, the between-group differences were small with mean knowledge score (out of 8) only varying from just under to just over three between groups. This suggests approaches designed to increase understanding would be beneficial for all.

Our study benefited from a large sample that was population representative with respect to age, region, education and social grade. We used mostly validated items which allowed comparison with previous studies. However, it also has some limitations. Online recruitment may have systematically excluded some groups from taking part and may limit the generalisability of the findings to all parts of the wider population. The item assessing awareness of HPV primary screening was developed for this survey and may not have been clearly understood by participants as its face validity was not assessed prior to use. Low awareness of primary screening is consistent with findings from qualitative research^27,28^ but more detailed exploration of women's understanding of screening processes and possible results would be useful. Almost a third of participants (30.3%) chose not to report their ethnicity. YouGov provide participants with an option to skip sensitive questions, including about ethnicity, in line with data protection regulations. This limits the conclusions we can draw about ethnic differences in knowledge. This would also benefit from more in-depth exploration in future research. Finally, we did not collect data on time since last screen among participants who were at least five years overdue. This ‘overdue’ group could therefore be very heterogeneous with respect to screening knowledge, attitudes and experience. However, the similar outcomes in those who were overdue and those who had never been screened (Tables 3 and 4) suggest that it is being up to date with screening that predicts better knowledge and awareness rather than the degree to which someone is overdue.

## Conclusion

Although awareness of HPV is increasing in Britain, many women still lack even a basic understanding of the virus and the role of HPV testing in cervical screening. Raising knowledge and awareness would not only increase women's ability to make informed choices about screening but would also likely reduce the adverse psychological consequences of an HPV-positive result. A good understanding of HPV will also be critical to ensuring acceptability of the lengthening of screening intervals that is likely to be introduced in England soon.

## Supplemental Material

sj-docx-1-msc-10.1177_09691413231205965 - Supplemental material for Awareness and knowledge about HPV and primary HPV screening among women in Great Britain: An online population-based survey Supplemental material, sj-docx-1-msc-10.1177_09691413231205965 for Awareness and knowledge about HPV and primary HPV screening among women in Great Britain: An online population-based survey by Jo Waller, Frances Waite and Laura Marlow in Journal of Medical Screening

sj-docx-2-msc-10.1177_09691413231205965 - Supplemental material for Awareness and knowledge about HPV and primary HPV screening among women in Great Britain: An online population-based survey Supplemental material, sj-docx-2-msc-10.1177_09691413231205965 for Awareness and knowledge about HPV and primary HPV screening among women in Great Britain: An online population-based survey by Jo Waller, Frances Waite and Laura Marlow in Journal of Medical Screening
