# Supplementary figures and images for: Awareness and knowledge about HPV and primary HPV screening among women in Great Britain: An online population-based survey
Source: J Med Screen. 2023 Oct 24;31(2):91–8. doi: 10.1177/09691413231205965 (PMC11083738; doi:10.1177/09691413231205965)

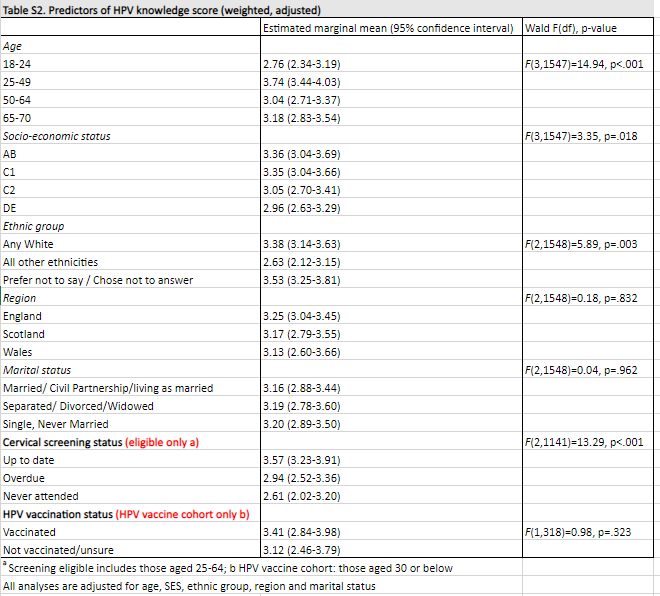

Supplement: sj-docx-1-msc-10.1177_09691413231205965 - Supplemental material for Awareness and knowledge about HPV and primary HPV screening among women in Great Britain: An online population-based survey [file sj-docx-1-msc-10.1177_09691413231205965.docx]
