# Supplementary material for: Awareness and knowledge about HPV and primary HPV screening among women in Great Britain: An online population-based survey
Source: J Med Screen. 2023 Oct 24;31(2):91–8. doi: 10.1177/09691413231205965 (PMC11083738; doi:10.1177/09691413231205965)
Supplement: sj-docx-2-msc-10.1177_09691413231205965 - Supplemental material for Awareness and knowledge about HPV and primary HPV screening among women in Great Britain: An online population-based survey [file sj-docx-2-msc-10.1177_09691413231205965.docx]

**Coding procedure**

FW carried out the first round of coding. The coding framework was developed using a pre-written framework from a previous study (Low et al., 2012) and inductively adding new codes. FW developed the coding framework as coding was carried out, including combining codes that were not mentioned frequently. ‘Coding frame v3’ was developed at the end of the coding process.

FW sent the coding framework to JW and LM for feedback with one example for each code. From this feedback, FW combined two codes which were overlapping and separated STI/STD and virus/ infection as two separate codes.

LM double coded 200 cases. Kappa was above 0.9 for each of the codes 1-7, so it was decided that we did not need to double code the whole sample.

**References**

Low, E. L., Simon, A. E., Lyons, J., Romney-Alexander, D., & Waller, J. (2012). What do British women know about cervical cancer symptoms and risk factors? *European Journal of Cancer*, *48*(16), 3001-3008. <https://doi.org/https://doi.org/10.1016/j.ejca.2012.05.004>
